# For 50 years, his test has been saving museum artifacts

**DOI:** 10.1021/acscentsci.5c02408

**Published:** 2026-01-12

**Authors:** Rachel Brazil

## Abstract

Andrew Oddy looks back on his career as Keeper of Conservation at the British Museum and the exposure test that bears his name.

## Graphical abstract

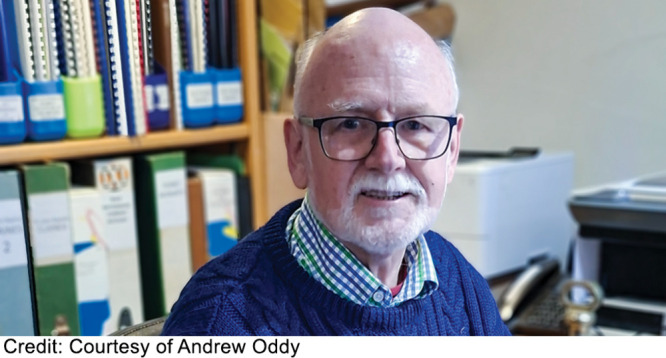

In 1966, after a year working at the former UK chemical giant Imperial Chemical Industries, Andrew Oddy realized that working in the chemical industry was not for him. Flicking through the magazine *Chemistry in Britain,* he saw the word *museum* in a job advertisement. “It was a bit of serendipity,” says Oddy, who had always loved archeology alongside chemistry and was attracted to the idea of working as a scientist in a museum.

**Figure:**
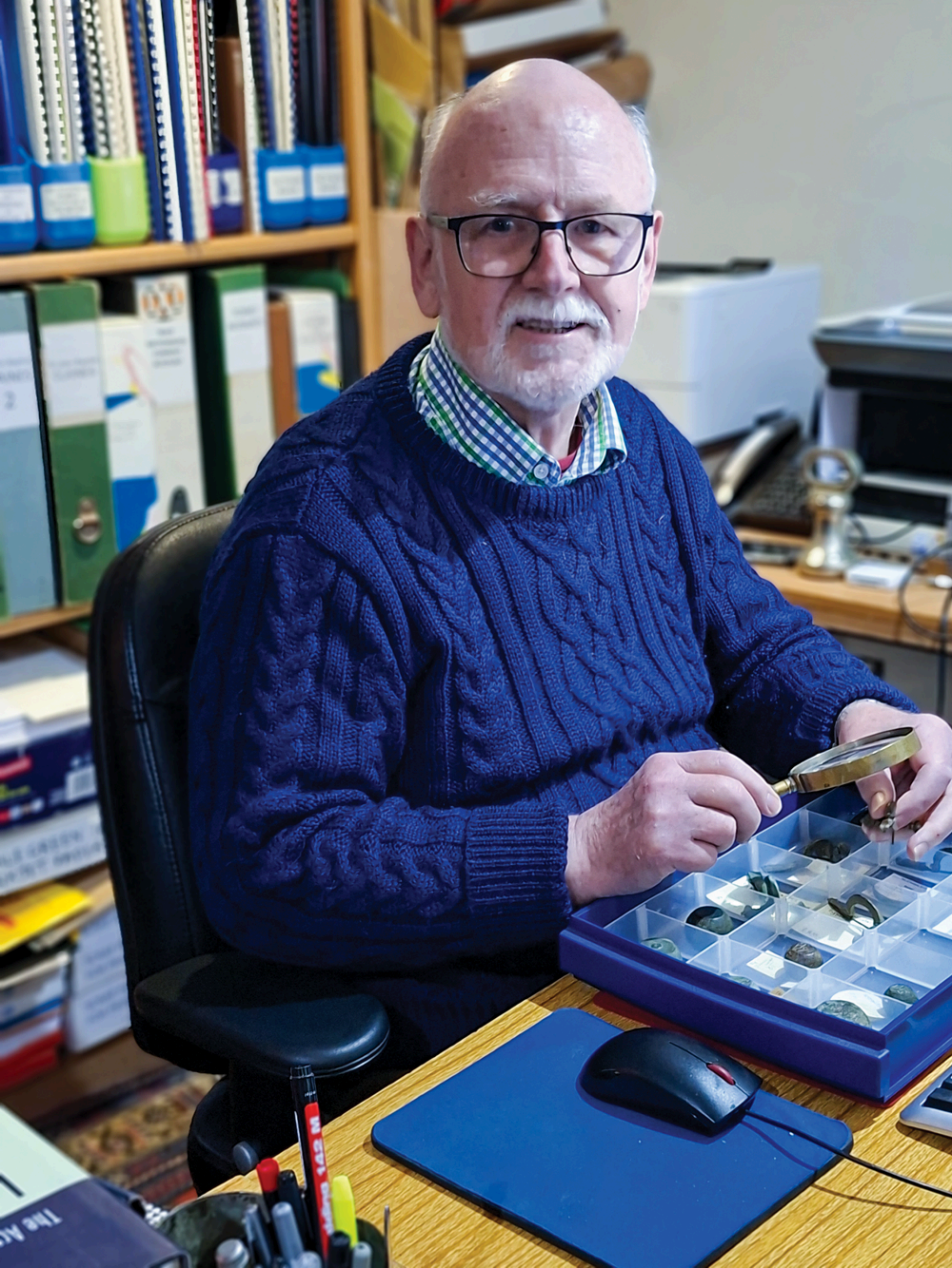
Andrew Oddy examines late Roman brooches found in Britain. Worn by army officers and government officials, the “tail” of each brooch has a specific decoration, but the meaning is unknown. Credit: Courtesy of Andrew Oddy.

He didn’t get that job, but he impressed the interview panel. Very soon after, one panel member offered him a position at the British Museum, where he spent the rest of his career, rising to Keeper of the Department of Conservation, a position he held until he retired in 2002.

Today, he is known in conservation circles for developing the namesake Oddy test, which celebrates its 50-year anniversary in 2025. The accelerated aging method helps museums establish whether the materials they use in display cases might damage metal artifacts. To conduct the test, scientists place a strip of metal in a sealed container with the test material—for example, a display case’s wood, fabric, or paint—and simulate how an artifact made of the same metal would corrode over a long period of time.

The Oddy test has become standard practice when designing new exhibitions. Some museums have further developed the test to make it quicker, more quantitative, and less subjective. Others are looking to broaden the test to other materials such as paper or plastic.

Researchers at New York City’s Metropolitan Museum of Art are trying to standardize and optimize the test as part of their Oddy Benchmarking Project. They’re applying a machine learning model to interpret results and are analyzing the metal strips to better characterize the corrosion products they form.

Rachel Brazil spoke to Oddy about how the test came about and some of the sticky problems he encountered during his career that led to conservation solutions. This interview has been edited for length and clarity.

## Why and how did you develop a test to prevent damage to museum artifacts on display?

The director of the Wallace Collection, another London museum, approached my boss to say that some Victorian and Georgian gold snuffboxes that they’d recently put out on exhibition had started to tarnish. He asked me to have a look.

They were tarnishing where there were pieces of jewelry set into the gold—where the gold was less pure and had enough silver to start blackening. We cut out a section of the display case’s materials, hardwood and plywood and a textile, and we did exposure tests.

To our utter amazement, it was the solid mahogany that was causing the blackening. We concluded that the wood for the showcase had been treated with either a fire retardant or possibly an insecticide treatment.

**Figure:**
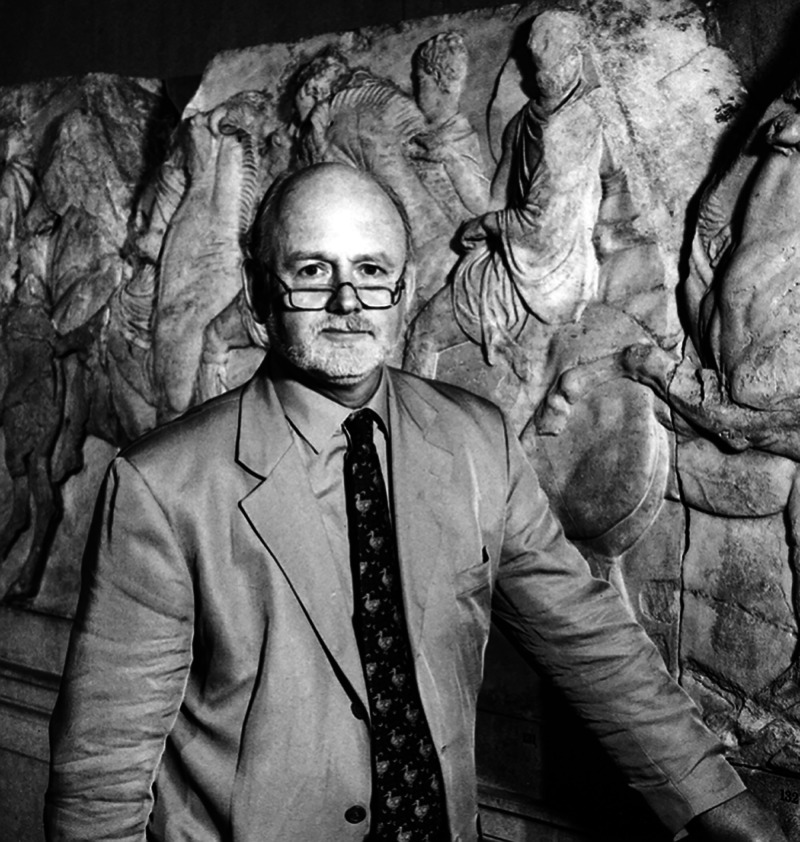
Andrew Oddy, at the British Museum in 2002, stands in front of one of the Elgin Marbles, originally part of the Parthenon in Athens. Credit: The British Museum.

## How did the test you designed work?

Because we were mostly interested in the deterioration of metals, we made a whole batch of little 1 cm^2^ pieces of thick silver foil, copper foil, and lead foil.

To do a test, we used to put a metal piece in a 250 mL flask covered with a ground glass stopper, and we put in moist cotton wool in a little test tube, so that the moisture didn’t actually have contact with the test piece.

We put [the whole assembly] in an oven at 60 °C for 28 days. And then we got the metal pieces out and looked at them for any corrosion.

If we saw slight or no corrosion, I think we passed it for use with temporary exhibitions. If we saw heavy corrosion, we said “Don’t use it.” But it was very subjective.

## How did the test become more widely used?

At about that time, we’d just put on a new exhibition of silver, and they lined the showcases with green felt. We took a sample of that green felt and did an exposure test, and it caused silver to corrode in a very short time.

So we started regularly testing things that were going in our own showcases. Soon after we got it going on a regular basis, we started providing a testing service for other museums.

## What other issues did you deal with during your career at the British Museum?

In the mid-1970s, our Egyptian department had a problem with some of its limestone sculptures because they’d been stored in basements for 200 or 300 years. Into the 20th century, the museum was warmed by coal fires, so stuff got terribly, terribly dirty.

The department started a program of washing the sculptures. They consulted the people at the Louvre, who were washing theirs with great success. [But when our department tried the Louvre’s technique], within a few minutes of going into the water, the surface began to lift off. They drained the tank and rushed over to us, and we started trying to investigate—part of normal life in a large museum!

It turned out that the sculptures at the Louvre had fortuitously all come from a quarry with almost no impurities in the limestone—very low amounts of acid-insoluble residue and salt content. Hence the stone washed safely, but this was pure luck.

We started testing our limestones by taking a little drilling of half a gram. We found out that if the acid-insoluble residue and the salt content were in the range 2–4%, it meant there was a lot of sulfate on the surface of the sculpture and you couldn’t wash it [without causing damage].

Modern industrial atmospheres convert the surface of limestone from calcium carbonate to calcium sulfate. The calcium sulfate crystals are larger, so it is very loosely adhered to the surface, and washing causes it to lift off.

**Figure:**
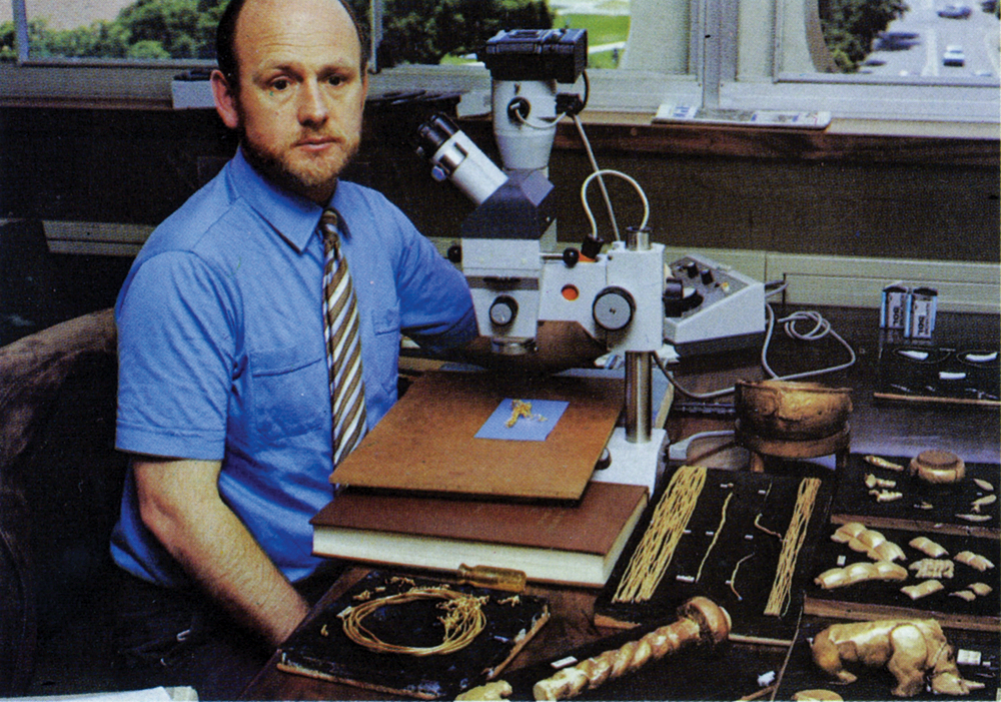
Andrew Oddy, on a 1983 visit to the University of Pretoria, examines gold objects (1220–90 CE) from the Mapungubwe Kingdom to provide conservation advice. Credit: Courtesy of the University of Pretoria: Mapungubwe Archive.

## Since your retirement, what have you focused on?

In the 1970s, I became interested in the Middle East and discovered that the earliest Islamic coinage of the seventh century CE in Greater Syria was very little studied. Since then, I have published numerous papers on aspects of the coinage, studying individual mints of towns in this area and proposing new mints from evidence derived from the coins. I’ve kept up this research since I retired, along with a bit of family history.

## Fifty years on, how do you feel about having the Oddy test named after you?

Now, I didn’t call it the Oddy test, because, in science, you can’t name things after yourself. But I admit to being chuffed.

I was at a conservation conference in the US when a young conservator looked at my name badge and said “Are you *the* Oddy test?”

*Rachel Brazil is a freelance contributor to* Chemical & Engineering News, *an independent news publication of the American Chemical Society.*

